# Emergency Allotments in SNAP and Food Hardship Among Households With Children

**DOI:** 10.1001/jamanetworkopen.2024.28680

**Published:** 2024-08-16

**Authors:** Anna E. Austin, Rebeccah L. Sokol

**Affiliations:** 1 Department of Health Behavior, Gillings School of Global Public Health, University of North Carolina at Chapel Hill; 2 Injury Prevention Research Center, University of North Carolina at Chapel Hill; 3 School of Social Work, University of Michigan, Ann Arbor; 4 Department of Health Behavior and Health Education, School of Public Health, University of Michigan, Ann Arbor

## Abstract

**Importance:**

Households with children and minoritized racial and ethnic groups experience a disproportionate burden of food hardship. During the COVID-19 pandemic, the US federal government implemented emergency allotments in the Supplemental Nutrition Assistance Program (SNAP), increasing the amount of food purchasing assistance received by many participating households.

**Objective:**

To examine the association of implementing emergency allotments in SNAP with food hardship among households with children overall and for households with Black, Hispanic, and White children by comparing income-eligible households that did and did not participate in SNAP.

**Design, Setting, and Participants:**

This ecologic cross-sectional study used 2016-2022 National Survey of Children’s Health data and a difference-in-differences approach to compare changes in the risk of food hardship from before implementation of emergency allotments in SNAP (2016-2019) to during implementation (2020-2022). Households with children younger than 18 years and incomes 130% or less of the federal poverty level (FPL) in all 50 states and Washington, DC, were included.

**Exposure:**

Implementation of emergency allotments in SNAP.

**Main Outcome and Measures:**

The primary outcome was caregiver report of household food hardship during the past 12 months.

**Results:**

Of 44 753 households with incomes 130% or less of the FPL, a weighted 23.4% had Black children, 56.7% had White children, and 19.9% had children of other races. More than one-third of households (37.8%) had Hispanic children, and 31.8% had young children aged 0 to 5 years. The percentage of households that experienced food hardship decreased from 2016 to 2021 (from 62.9% to 48.2% among SNAP-participating households and from 44.3% to 38.9% among income-eligible nonparticipating households) but increased in 2022 (to 58.0% among SNAP-participating households and to 47.5% among nonparticipating households). Adjusting for confounders, implementing emergency allotments in SNAP was associated with a decreased risk of food hardship among SNAP-participating compared with nonparticipating households (risk ratio [RR], 0.88; 95% CI, 0.81-0.96). Implementing emergency allotments in SNAP was associated with a decreased risk of food hardship among SNAP-participating households with Hispanic (RR, 0.86; 95% CI, 0.72-1.02) and White (RR, 0.85; 95% CI, 0.76-0.94) children compared with nonparticipating households but not among households with Black children (RR, 1.04; 95% CI, 0.87-1.23).

**Conclusions and Relevance:**

In this ecologic cross-sectional study, implementing emergency allotments in SNAP was associated with a decreased risk of food hardship among households with children. Efforts are needed to ensure that all populations benefit from economic policies.

## Key Points

**Question:**

Were emergency allotments in the Supplemental Nutrition Assistance Program (SNAP) associated with a change in the risk of food hardship among households with children?

**Findings:**

In this cross-sectional study of 44 753 households with incomes 130% or less of the federal poverty level and with children younger than 18 years, implementing emergency allotments in SNAP was associated with a decreased risk of food hardship among SNAP-participating households compared with households that did not participate in SNAP. This decreased risk was observed overall and for households with Hispanic and White children but not for households with Black children.

**Meaning:**

These results suggest that emergency allotments in SNAP may have contributed to a decreased risk of food hardship among households with children and indicate that efforts are needed to ensure that all populations benefit from economic policies.

## Teaser

This cross-sectional study examines the association of implementing emergency allotments in SNAP with food hardship among households with children overall and by race and ethnicity.

## Introduction

Food insecurity, or limited access to adequate food due to lack of money or resources, is a critical public health issue in the US. In 2022, more than 44 million people in the US lived in a household that experienced food insecurity.^1^ Importantly, households with children (17%) are more likely to experience food insecurity than households without children (11%).^1^ Due to structural racism and the systems that create and sustain inequitable resource distributions across populations,^2,3^ experiences of food insecurity are more than twice as high among households headed by Black (22%) and Hispanic (21%) individuals than among households headed by White individuals (9%).^1^

Limited access to food has health implications for both adults and children, increasing the risk for poor physical health and stress, anxiety, and depressive symptoms.^4,5,6,7,8,9^ Among children, early experiences of food insecurity are associated with an increased risk of poor physical and mental health across the life course, including during childhood, adolescence, and adulthood.^7,8,10,11,12,13^ Racial and ethnic disparities in experiences of food insecurity mean that these potential adverse health outcomes may disproportionately affect individuals belonging to minoritized racial and ethnic groups.^2,3^

The Supplemental Nutrition Assistance Program (SNAP) is the largest programmatic and policy strategy to alleviate food insecurity in the US. Currently, more than 20 million low-income households participate in SNAP and receive a monthly benefit to assist with the cost of purchasing food.^14^ Prior research has shown that SNAP participation is associated with reductions in poverty and food insecurity,^15,16,17,18^ particularly among households with children, and improved physical and mental health for children and adults.^18,19,20,21^

At the onset of the COVID-19 pandemic, amid concerns that rising unemployment would contribute to an increased need for food purchasing assistance, the federal government implemented emergency allotments in SNAP. Beginning in March 2020 under the Families First Coronavirus Response Act, states could request waivers allowing them to provide all SNAP-participating households with the maximum monthly benefit possible given household size.^22^ By May 2020, all states had requested and received approval to implement these emergency allotments.^23^ The federal government issued additional guidance in April 2021, specifying that all SNAP-participating households were to receive a minimum of $95 in monthly SNAP benefits, regardless of the maximum monthly benefit possible for their household size. Recent research has shown that emergency allotments decreased the risk of food insecurity among adults participating in SNAP in the early months of the pandemic.^24^ At the federal level, emergency allotments in SNAP ended in March 2023, but some states chose to end them early in 2021 or 2022.^22^ Initial evidence has indicated that the percentage of adults reporting that their household did not have enough food to eat increased after these states ended emergency allotments early, suggesting that emergency allotments may have protected against increases in difficulties affording food during the pandemic.^25^ The association of emergency allotments in SNAP with food hardship among households with children, a particularly vulnerable population, has not been examined. The aim of this study was to assess whether implementing temporary emergency allotments in SNAP was associated with a change in the risk of food hardship among households with children.

## Methods

### Data Source and Study Population

This ecologic cross-sectional study used population-representative data from the 2016-2022 National Survey of Children’s Health (NSCH), an annual survey designed to produce national and state estimates of child and family health and well-being. This study was considered exempt from approval and the need for informed consent due to the use of publicly available data by the institutional review board at the University of North Carolina. This study follows the Strengthening the Reporting of Observational Studies in Epidemiology (STROBE) reporting guideline for cross-sectional studies.

For the NSCH, the US Census Bureau contacts a random sample of households each year by mail to identify households with at least 1 child younger than 18 years.^26^ For households with multiple children, 1 child is randomly selected to be the survey subject. An adult member of the household (ie, caregiver) who is familiar with the child’s health, usually a parent, completes the survey on paper or online in English or Spanish.^26^ Surveys are completed from June to January of each year (eg, 2021 surveys are completed June 2021 to January 2022). In this study, we included households with incomes 130% or less of the federal poverty level (FPL), the income limit for SNAP eligibility at the federal level.^27^

### Exposure and Outcome

The NSCH assesses food hardship using a single question that asks caregivers, “Which of these statements best describes your household’s ability to afford the food you needed during the past 12 months?” Response options include “We could always afford enough to eat good nutritious meals,” “we could always afford enough to eat but not always the kinds of food we should eat,” “sometimes we could not afford enough to eat,” and “often we could not afford enough to eat.” We considered the latter 3 responses, which capture both the quality (ie, the household not being able to afford the kinds of foods they should) and quantity (ie, the household’s ability to afford enough food) of food,^28^ to indicate household food hardship. The NSCH also asks caregivers whether at any time during the past 12 months, even for 1 month, anyone in their family received food stamps or SNAP benefits, with response options of yes or no. We compared food hardship for households participating in SNAP and income-eligible households not participating in SNAP from before (2016-2019) to during (2020-2022) implementation of emergency allotments.

### Confounders

We created a conceptual diagram to identify factors associated with both SNAP participation and household food hardship (eFigure and eTable 1 in Supplement 1). Prior research has indicated that there are systematic differences between income-eligible households that do and do not participate in SNAP. Among income-eligible households, those that participate in SNAP experience more severe food hardships and have lower incomes and fewer economic resources than nonparticipating households.^29,30,31^ To account for factors that are likely associated with both the likelihood that households participate in SNAP and household food hardship, we adjusted analyses for household receipt of cash assistance from a government program in the past 12 months, employment status of adults in the household, and household income relative to the FPL. In addition, given that food hardships and poverty disproportionately affect minoritized racial and ethnic populations,^1,14,32^ we adjusted analyses for caregiver-reported child race and ethnicity (Black, Hispanic, White, or other [including American Indian or Alaska Native, Asian Indian, Chinese, Filipino, Guamanian or Chamorro, Japanese, Korean, Native Hawaiian, Other Asian, Pacific Islander, Samoan, and Vietnamese]). Caregiver race and ethnicity are not captured in the NSCH. We also adjusted analyses for time-varying state-level economic policies that may contribute to household food hardship and thus SNAP participation, including state minimum wage,^33,34^ refundable Earned Income Tax Credit rate,^35,36^ maximum Temporary Assistance for Needy Families benefits for a family of 3,^37^ and Medicaid expansion.^37,38^

### Statistical Analysis

We used a difference-in-differences approach to compare changes in the risk of food hardship from before to during implementation of emergency allotments in SNAP among income-eligible households that did and did not participate in SNAP. We constructed a log-binomial regression model to calculate risk ratios (RRs) and corresponding 95% CIs. We specified the model as follows:

g(E[Y_ist_]) = β_0_ + β_1_*SNAP_i_ + β_2_*EA_t_ + β_3_*SNAP_i_*EA_t_ + β_4_ X_st_ + β_5_ X_i_,

where *i* indexes households, *s* indexes states, and *t* indexes time; *Y_ist_* is a binary indicator for household food hardship for household *i* in state *s* at time *t*, and *g* is the log-link function defined as g(u) = ln(u); *SNAP_i_* is a binary indicator for whether household *i* participated in SNAP in the past 12 months; and *EA_t_* is a binary indicator for whether the survey responses corresponded to years when emergency allotments in SNAP were implemented (ie, 2020-2022). The coefficient for the interaction term between *SNAP_i_* and *EA_t_*, *β_3_*, is the difference-in-differences estimator and indicates whether the change in the risk of food hardship from before to during implementation of emergency allotments in SNAP differs for income-eligible households that did and did not participate in SNAP. Negative values of *β_3_*, corresponding to RRs of less than 1.0, indicate that the risk of food hardship decreased among SNAP-participating households compared with nonparticipating households from before to during implementation of emergency allotments (ie, implementing emergency allotments in SNAP was associated with a decreased risk of food hardship among SNAP-participating households relative to nonparticipating households). Positive values of *β_3_*, corresponding to RRs greater than 1.0, indicate that the risk of food hardship increased among SNAP-participating households compared with nonparticipating households from before to during implementation of emergency allotments. A *β_3_* value of 0, corresponding to an RR of 1.0, indicates no difference in the change in the risk of food hardship among SNAP-participating households compared with nonparticipating households from before to during implementation of emergency allotments. The variable *X_i_* is a vector of household characteristics, and *X_st_* is a vector of time-varying state economic policies, as described above, included to adjust for potential confounding. Given persistent racial and ethnic disparities in food hardships,^1,2,3^ we constructed models overall and separately for households with Black, Hispanic, and White children.

A key assumption in the difference-in-differences approach is the parallel trends assumption, or that in the absence of emergency allotments in SNAP, trends in food hardship would have been similar between SNAP-participating and nonparticipating households. Our assessment of the parallel trends assumption indicated that it was met, conditional on potential confounders (eMethods in Supplement 1).

**Figure 1.**
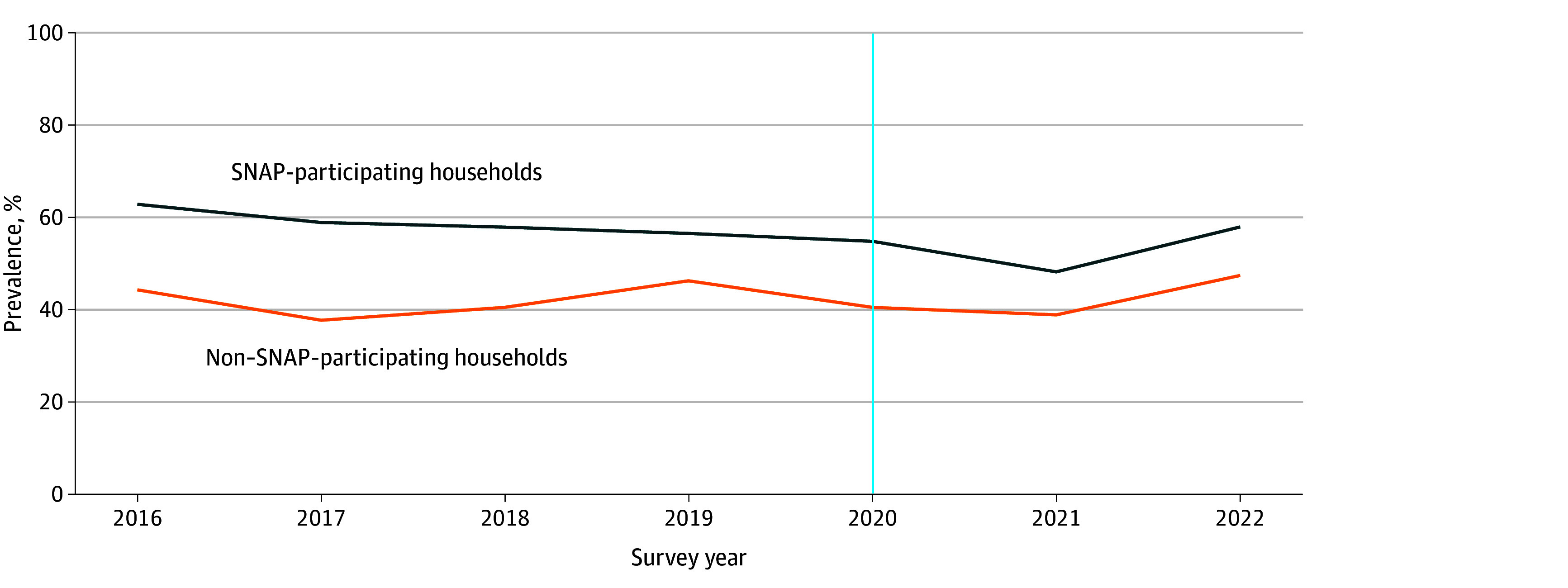
Prevalence of Food Hardship in the Past 12 Months Among Households With Income of 130% or Less of the Federal Poverty Level, Stratified by Supplemental Nutrition Assistance Program (SNAP) Participation Total number of households assessed was 46 789. The vertical line indicates the onset of the COVID-19 pandemic and emergency allotments in SNAP.

We conducted multiple sensitivity analyses. First, because 10 states ended emergency allotments early in 2021 (Arkansas, Florida, Idaho, Nebraska, North Dakota, Missouri, Michigan, Mississippi, South Dakota, and Tennessee) and 7 in 2022 (Alaska, Arizona, Georgia, Indiana, Iowa, Kentucky, and Wyoming), we conducted sensitivity analyses entirely excluding these states. Second, given the retrospective nature of caregiver reports of household food hardship (ie, during the past 12 months) and that the NSCH is conducted June to January of each year, we conducted sensitivity analyses that excluded 2020 given that some 2020 survey responses may have captured food hardship only experienced before implementation of emergency allotments (eg, survey responses in June 2020 could capture food hardship experienced any time from June 2019 to June 2020). Third, given a small increase in the percentage of income-eligible non–SNAP-participating households experiencing food hardship in 2019, compared to 2018, that was not observed among income-eligible SNAP-participating households (Figure 1), we conducted sensitivity analyses excluding 2019. Fourth, under broad-based categorical eligibility (BBCE), states can expand SNAP eligibility by increasing the income limit from 130% to up to 200% of the FPL and/or eliminating the asset test used to determine eligibility.^39^ We conducted sensitivity analyses that additionally adjusted for state adoption of these policies. Fifth, we conducted sensitivity analyses among households with incomes 200% or less of the FPL given that some states increased the income limit up to 200% of the FPL under BBCE. Finally, we conducted sensitivity analyses using low or very low food insufficiency, defined as responses of “sometimes we could not afford enough to eat” or “often we could not afford enough to eat” during the past 12 months,^27,40,41^ as the outcome.

We conducted analyses using SAS, version 9.4 (SAS Institute Inc) and Stata, version 17.0 (StataCorp LLC) statistical software. We used NSCH sampling weights to account for the probability of household selection and nonresponse and to generate population-representative results. For analyses among households with Black, Hispanic, and White children, we used the subpopulation estimation in Stata to ensure correct calculation of standard errors. To interpret results, we relied on the magnitude of the adjusted RR and the width and location of the corresponding 95% CIs, in alignment with guidance from the American Statistical Association.^42,43^

## Results

Of 44 753 households with incomes 130% or less of the FPL, a weighted 23.4% had Black children, 56.7% had White children, and 19.9% had children of other races. More than one-third of households (37.8%) had Hispanic children, and 31.8% had young children aged 0 to 5 years. From 2016 to 2022, the percentage of households with children that experienced food hardship during the past 12 months was consistently higher among those that participated in SNAP compared with those that did not (Figure 1; eTable 2 in Supplement 1). From 2016 to 2021, the percentage of households that experienced food hardship decreased from 62.9% to 48.2% among those participating in SNAP and from 44.3% to 38.9% among those not participating in SNAP. The percentage of households that experienced food hardship increased in 2022 to 58.0% among SNAP-participating households and to 47.5% among nonparticipating households. Over the entire study period, a higher percentage of SNAP-participating households compared with nonparticipating households received cash assistance from a government program (18.1% vs 3.1%), did not have an employed adult (30.8% vs 14.0%), had incomes between 0% and 65% of the FPL (53.5% vs 38.5%), and had Black children (30.3% vs 15.1%) (Table 1).

**Table 1.** Characteristics of Households With Children Younger Than 18 Years and Incomes of 130% or Less of the FPL, 2016-2022 National Survey of Children’s Health (N = 44 753)

Characteristic	SNAP-participating households (n = 20 474)		Non–SNAP-participating households (n = 24 279)	
	No.	% (95% CI)^a^	No.	% (95% CI)^a^
Food hardship in past 12 mo				
No	8146	43.1 (41.7-44.6)	14 322	57.7 (56.3-59.2)
Yes	12 196	56.9 (55.4-58.3)	9812	42.3 (40.8-43.7)
Receipt of cash assistance from a government program in past 12 mo				
No	16 200	81.9 (80.7-83.1)	23 609	96.9 (96.4-97.5)
Yes	3599	18.1 (16.9-19.3)	618	3.1 (2.5-3.6)
Caregiver employment status				
Not employed	6459	30.8 (29.5-32.2)	2900	14.0 (13.0-15.0)
Employed	13 216	69.2 (67.8-70.5)	20 580	86.0 (85.0-87.0)
Household income relative to the FPL, %				
0-65	10 287	53.5 (52.0-54.9)	9526	38.5 (37.1-39.9)
66-130	10 187	46.5 (45.1-48.0)	14 753	61.5 (60.1-62.9)
Child race				
Black	4394	30.3 (29.1-31.6)	2560	15.1 (14.2-16.1)
White	12 164	51.5 (50.0-52.9)	17 016	63.1 (61.7-64.5)
All other races^b^	3916	18.2 (17.1-19.4)	4703	21.8 (20.5-23.1)
Child ethnicity				
Hispanic	4620	34.3 (32.8-35.8)	5520	41.2 (39.7-42.7)
Non-Hispanic	15 854	65.7 (64.2-67.2)	18 759	58.8 (57.3-60.3)

Abbreviations: FPL, federal poverty level; SNAP, Supplemental Nutrition Assistance Program.

^a^ Weighted percentages based on National Survey of Children’s Health sampling weights.

^b^ All other races include American Indian or Alaska Native, Asian Indian, Chinese, Filipino, Guamanian or Chamorro, Japanese, Korean, Native Hawaiian, Other Asian, Pacific Islander, Samoan, and Vietnamese.

**Figure 2.**
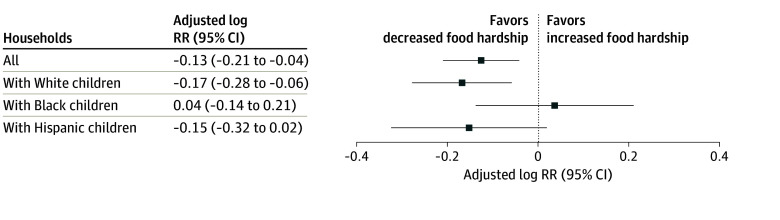
Change in Risk of Food Hardship From Before (2016-2019) to During (2020-2022) Implementation of Emergency Allotments in the Supplemental Nutrition Assistance Program (SNAP) Among Income-Eligible Participating Households With Children Compared With Nonparticipating Households, 2016-2022 National Survey of Children’s Health Log risk ratios (RRs) were adjusted for household receipt of cash assistance from a government program in the past 12 months; employment status of adults in the household; household income relative to the federal poverty level; child race and ethnicity; and state minimum wage, refundable Earned Income Tax Credit rate, maximum Temporary Assistance for Needy Families benefit for a family of 3, and Medicaid expansion.

Adjusting for potential confounders, implementing emergency allotments in SNAP was associated with a decreased risk of food hardship among SNAP-participating households with children compared with income-eligible nonparticipating households with children (RR, 0.88; 95% CI, 0.81-0.96) (Figure 2; Table 2). Results were similar in sensitivity analyses excluding the 17 states that ended emergency allotments in 2021 or 2022 (RR, 0.89; 95% CI, 0.78-0.98), excluding 2020 given the retrospective nature of caregiver reports of food hardship (RR, 0.88; 95% CI, 0.80-0.96), excluding 2019 given the small increase in the percentage of non–SNAP-participating households reporting food hardship (RR, 0.86; 95% CI, 0.79-0.94), and additionally adjusting for state expansion of SNAP eligibility under BBCE (RR, 0.90; 95% CI, 0.83-0.97) (eTable 3 in Supplement 1). The reduction in the risk of food hardship was slightly smaller in sensitivity analyses that included households with incomes 200% or less of the FPL (RR, 0.92; 95% CI, 0.86-0.98). In sensitivity analyses with food insufficiency as the outcome, results were consistent with a decreased risk of food insufficiency among SNAP-participating households with children compared with income-eligible nonparticipating households with children during implementation of emergency allotments (RR, 0.88; 95% CI, 0.68-1.14). While the magnitude of the RRs and most of the 95% CI was consistent with a decreased risk, the 95% CI was wider, which may have been due to the lower prevalence of food insufficiency compared with food hardship among households with incomes 130% or less of the FPL (13.9% vs 49.3%), and included the null.

**Table 2.** Change in Risk of Food Hardship From Before (2016-2019) to During (2020-2022) Implementation of Emergency Allotments in SNAP Among Income-Eligible Participating Households With Children Compared With Nonparticipating Households, 2016-2022 National Survey of Children’s Health

Food hardship among households with ≤130% FPL	Adjusted difference-in-differences estimator, RR for interaction term (95% CI)^a^
All households	
Participating in SNAP	0.88 (0.81-0.96)
Not participating in SNAP	1 [Reference]
Households with Black children	
Participating in SNAP	1.04 (0.87-1.23)
Not participating in SNAP	1 [Reference]
Households with Hispanic children	
Participating in SNAP	0.86 (0.72-1.02)
Not participating in SNAP	1.00 [Reference]
Households with White children	
Participating in SNAP	0.85 (0.76-0.94)
Not participating in SNAP	1 [Reference]

Abbreviations: FPL, federal poverty level; RR, risk ratio; SNAP, Supplemental Nutrition Assistance Program.

^a^ Adjusted for household receipt of cash assistance from a government program in the past 12 months, employment status of adults in the household, household income relative to the FPL, child race and ethnicity, and state minimum wage, refundable Earned Income Tax Credit rate, maximum Temporary Assistance for Needy Families benefit for a family of 3, and Medicaid expansion.

In analyses stratified by child race and ethnicity, implementing emergency allotments in SNAP was associated with a decreased risk of food hardship among SNAP-participating households with Hispanic (RR, 0.86; 95% CI, 0.72-1.02) and White (RR, 0.85; 95% CI, 0.76-0.94) children compared with nonparticipating households with Hispanic children and White children, respectively (Figure 2; Table 2). Implementing emergency allotments in SNAP was not associated with a change in the risk of food hardship among SNAP-participating households with Black children compared with income-eligible nonparticipating households with Black children (RR, 1.04; 95% CI, 0.87-1.23). Notably, households with Black children were disproportionately represented among those with lower incomes (28.0% among households with incomes between 0% and 65% of the FPL vs 19.3% among households with incomes between 66% and 130% of the FPL) compared with households with White children (52.3% among households with incomes between 0% and 65% of the FPL vs 60.7% among households with incomes between 66% and 130% of the FPL) (eTable 4 in Supplement 1).

## Discussion

The results of this ecologic cross-sectional study show that emergency allotments in SNAP were associated with a decreased risk of food hardship among households with children that participated in SNAP compared with income-eligible households with children that did not participate in SNAP. This decreased risk is notable given the unprecedented health and economic hardship that occurred during the COVID-19 pandemic.^44,45,46,47^ Importantly, we observed a decreased risk among households with Hispanic and White children but not among households with Black children, suggesting that the potential benefits of emergency allotments were not equally realized for all populations.

Recent studies have shown decreases in the risk of difficulty affording food among SNAP-participating households during implementation of emergency allotments.^24,25^ Our results are consistent with these studies and add a focus on households with children, a high-risk population for food hardship.^1^ Collectively, these results reinforce the critical role of SNAP in mitigating food hardship, particularly during economic crises.^48^ In addition to emergency allotments in SNAP, other economic supports were implemented during the COVID-19 public health emergency, including an expanded Child Tax Credit (CTC) in 2021.^49^ Recent research has shown that the expanded CTC was associated with reductions in the likelihood that households with children did not have enough food to eat.^40,41,50^ Importantly, the expanded CTC was available to all low-income households with children, including those that did and did not participate in SNAP, and thus likely did not systematically affect our results.

We observed a decreased risk of food hardship among SNAP-participating households with Hispanic children and White children compared with income-eligible nonparticipating households during implementation of emergency allotments. However, we did not observe this decreased risk among households with Black children. Food environments are racially segregated, with fewer grocery stores, limited access to healthy food options, and higher food prices in predominantly Black communities.^51,52^ In addition, among SNAP-participating households with children, Black households report needing a larger increase in SNAP benefits to afford enough food than White households ($51 vs $42 more per week).^53^ Segregated food environments, as well as the larger gap between the amount of SNAP benefit received and the amount needed, may have limited the potential for emergency allotments to contribute to reductions in food hardship among households with Black children. Furthermore, in our data, households with Black children were disproportionately represented among those with incomes 0% to 65% of the FPL. In SNAP, monthly benefit amounts are proportional to income such that households with lower incomes receive a higher benefit up to a maximum benefit possible given household size.^54^ SNAP-participating households that were already receiving the maximum benefit possible did not experience an increase in food purchasing assistance under emergency allotments from March 2020 to April 2021. Only those with monthly benefits less than $95 experienced an increase in food purchasing assistance after April 2021.^55^ If more households with Black children than households with Hispanic or White children were already receiving the maximum benefit possible, they may not have received the same level of economic support from emergency allotments. Existing research has confirmed that among SNAP-participating households with children, Black households were more likely than White households to already be receiving the maximum benefit possible prior to implementation of emergency allotments.^56^ This research has also shown that Black households experienced a smaller increase in monthly benefit amount during implementation of emergency allotments, including when the minimum monthly benefit was increased to $95 for all households, than White households.^56^

### Limitations

This study has several limitations. First, though we adjusted multivariable analyses for a comprehensive set of confounders, there is potential for unmeasured or residual confounding specifically due to unmeasured or poorly measured factors that affect both SNAP participation and food hardship. Because income-eligible households that participate in SNAP are more likely to experience more severe food hardship and have fewer economic resources than those that do not participate in SNAP, this unmeasured or residual confounding would likely bias our results toward the null. Second, the NSCH measures food hardship over the past 12 months. There may have been issues with recall, though we do not expect that recall would have systematically differed for households that did and did not participate in SNAP. In addition, given the single report of food hardship over the past 12 months, we were unable to precisely align reports of food hardship with the month of emergency allotment implementation. Third, the NSCH did not include a validated measure of food hardship, such as the US Department of Agriculture’s 18-item measure of food insecurity.^28^ Fourth, the NSCH only collects information on child race and ethnicity, which may not be the same as the race and ethnicity of the child’s caregiver or other members of the household. Finally, given the lack of data at a monthly level, we were not able to examine the potential implications of changes in emergency allotment guidance in spring 2021.^55^ Future research is needed to examine whether ensuring a minimum monthly SNAP benefit of at least $95 for all participating households is associated with changes in the risk of food hardship.

## Conclusions

The results from this ecologic cross-sectional study suggest that policies that provide economic support to children and their families, such as emergency allotments in SNAP, may benefit population-level health. These results can be used to inform ongoing discussions regarding the potential need to permanently increase SNAP benefits.^57,58^ Future research is needed to examine the implications of the end of emergency allotments in SNAP on child and family food hardship and to understand strategies to ensure that the potential benefits of such economic support policies are realized for all populations.

## Notes

Supplement 1. **eFigure.** Conceptual Diagram **eTable 1.** Potential Confounders Included in the Multivariable Analyses **eMethods.** Parallel Trends Assumption **eTable 2.** Prevalence of Food Hardship in the Past 12 Months Among Households That Did and Did Not Participate in the Supplemental Nutrition Assistance Program (SNAP) and Had Incomes ≤130% of the Federal Poverty Level by Year **eTable 3.** Sensitivity Analyses for Change in Risk of Food Hardship From Before (2016-2019) to During (2020-2022) Implementation of Emergency Allotments in the Supplemental Nutrition Assistance Program (SNAP) for Income-Eligible Households With Children Participating in SNAP Compared to Income-Eligible Households With Children Not Participating in SNAP, 2016-2022 National Survey of Children’s Health **eTable 4.** Household Income Relative to the Federal Poverty Level by Child Race and Ethnicity, 2016-2022 National Survey of Children’s Health

Supplement 2.
Data Sharing Statement
