# Supplementary material for: Emergency Allotments in SNAP and Food Hardship Among Households With Children
Source: JAMA Netw Open. 2024 Aug 16;7(8):e2428680. doi: 10.1001/jamanetworkopen.2024.28680 (PMC11329880; doi:10.1001/jamanetworkopen.2024.28680)
Supplement: Supplement 1. — eFigure. Conceptual Diagram eTable 1. Potential Confounders Included in the Multivariable Analyses eMethods. Parallel Trends Assumption eTable 2. Prevalence of Food Hardship in the Past 12 Months Among Households That Did and Did Not Participate in the Supplemental Nutrition Assistance Program (SNAP) and Had Incomes ≤130% of the Federal Poverty Level by Year eTable 3. Sensitivity Analyses for Change in Risk of Food Hardship From Before (2016-2019) to During (2020-2022) Implementation of Emergency Allotments in the Supplemental Nutrition Assistance Program (SNAP) for Income-Eligible Households With Children Participating in SNAP Compared to Income-Eligible Households With Children Not Participating in SNAP, 2016-2022 National Survey of Children’s Health eTable 4. Household Income Relative to the Federal Poverty Level by Child Race and Ethnicity, 2016-2022 National Survey of Children’s Health [file jamanetwopen-e2428680-s001.pdf]

Supplemental Online Content

Austin AE, Sokol RL. Emergency allotments in SNAP and food hardship among households with children. *JAMA Netw Open*. 2024;7(8):e2428680. doi:10.1001/jamanetworkopen.2024.28680

eFigure. Conceptual Diagram

eTable 1. Potential Confounders Included in the Multivariable Analyses

eMethods. Parallel Trends Assumption

eTable 2. Prevalence of Food Hardship in the Past 12 Months Among Households That Did and Did Not Participate in the Supplemental Nutrition Assistance Program (SNAP) and Had Incomes ≤130% of the Federal Poverty Level by Year

eTable 3. Sensitivity Analyses for Change in Risk of Food Hardship From Before (2016-2019) to During (2020-2022) Implementation of Emergency Allotments in the Supplemental Nutrition Assistance Program (SNAP) for Income-Eligible Households With Children Participating in SNAP Compared to Income-Eligible Households With Children Not Participating in SNAP, 2016-2022 National Survey of Children's Health

eTable 4. Household Income Relative to the Federal Poverty Level by Child Race and Ethnicity, 2016-2022 National Survey of Children's Health

This supplemental material has been provided by the authors to give readers additional information about their work.

© 2024 Austin AE et al. *JAMA Network Open.*

eFigure. Conceptual Diagram

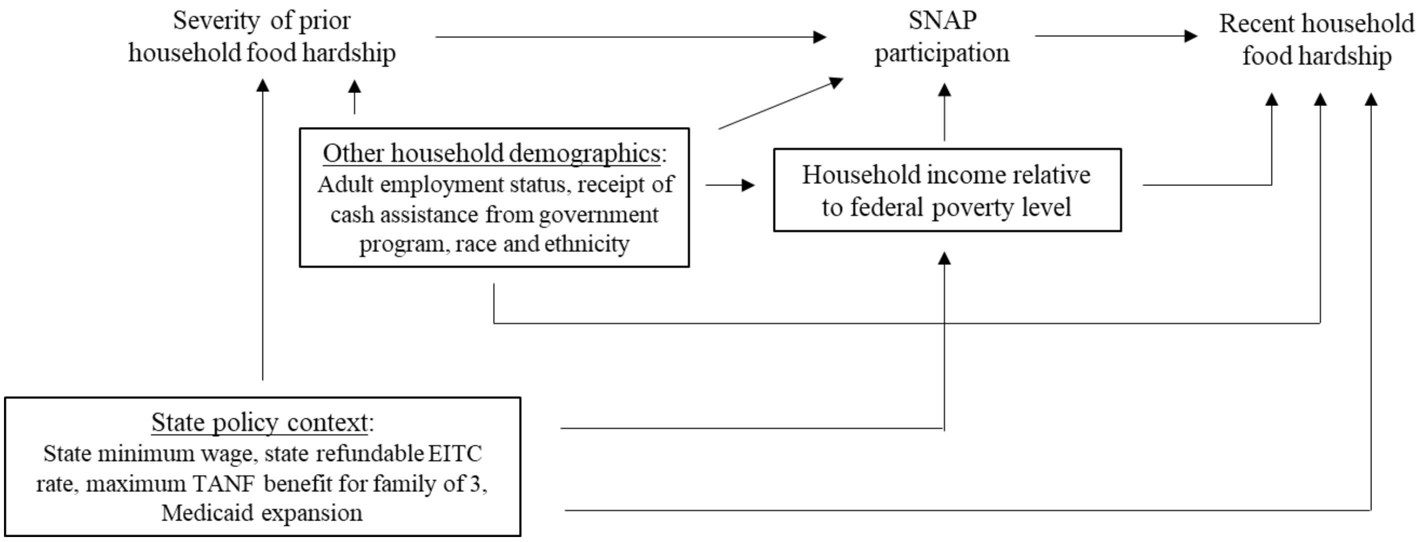

Note: Boxes denote variables adjusted for in multivariable analyses.

© 2024 Austin AE et al. *JAMA Network Open.*

eTable 1. Potential Confounders Included in Multivariable Analyses

Variable	Measure	Data source	Operationalized in analyses
Household receipt of cash assistance from a government program in the past 12 months	At any time during the past 12 months, even for one month, did anyone in your family receive cash assistance from a government welfare program?	National Survey of Children's Health	Yes vs. no
Employment status of adults in the household	At least one of the child's 2 primary caregivers in the household were employed during at least 50 of the past 52 weeks	National Survey of Children's Health	Yes (at least 1 primary caregiver employed) vs. no (neither of 2 primary caregivers employed)
Household income relative to the Federal Poverty Level	Household income relative to the Federal Poverty level calculated caregiver reported household income and household size during the prior calendar year	National Survey of Children's Health	0-65% of Federal Poverty Level vs. 66-130% of Federal Poverty Level
Caregiver reported child race	What is the child's race? [American Indian or Alaska Native; Asian Indian; Black or African American; Chinese; Filipino, Japanese; Guamanian or Chamorro; Korean; Native Hawaiian; Samoan; White; Other Asian; Other Pacific Islander; Vietnamese]	National Survey of Children's Health	Black or African American, White, all other races (American Indian or Alaska Native; Asian Indian; Chinese; Filipino, Japanese; Guamanian or Chamorro; Korean; Native Hawaiian; Samoan; Other Asian; Other Pacific Islander; Vietnamese)
Caregiver reported child ethnicity	Is the child of Hispanic, Latino, or Spanish origin? [No; Yes, Mexican, Mexican American, or Chicano; Yes, Puerto Rican; Yes, Cuban; Yes, another Hispanic, Latino, or Spanish origin]	National Survey of Children's Health	Hispanic vs. non-Hispanic
State minimum wage	State minimum wage in dollars	University of Kentucky National Welfare Database	Real value of state minimum wage; if state minimum wage was lower than the Federal minimum wage, the Federal minimum wage was used as the Federal minimum wage overrides the state if the state minimum wage is lower
State Refundable Earned Income Tax Credit rate	State Earned Income Tax Credit rate as a percentage of the Federal Earned Income Tax Credit	University of Kentucky National Welfare Database	State Earned Income Tax Credit rate as a percentage of the Federal Earned Income Tax Credit
State maximum Temporary Assistance for Needy Families benefits for a family of 3	Maximum monthly benefit for a 3-person family in dollars	University of Kentucky National Welfare Database	Real value of the maximum monthly benefit for a 3-person family
State Medicaid expansion	State Medicaid expansion status	University of Kentucky National Welfare Database	Yes vs. no

Note: For each state-level covariate, each child was assigned the value corresponding to their state of residence and year of National Survey of Children's Health participation. State-level covariates were time-varying.

© 2024 Austin AE et al. *JAMA Network Open.*

eMethods. Parallel Trends Assumption

A key assumption in the difference-in-differences approach is the parallel trends assumption, or that in the absence of temporary emergency allotments in SNAP, trends in food hardship would have been similar between households that did and did not participate in SNAP. To assess the parallel trends assumption, we examined whether trends in food hardship prior to implementation of emergency allotments in SNAP were similar between income-eligible households that did and did not participate in SNAP. To do so, we constructed a log-binomial regression model using data from the period before emergency allotments were implemented (i.e., 2016-2019). The model included an indicator for year, an indicator household SNAP participation, an interaction term between year and household SNAP participation, and all household- and state-level confounders. In this model, the coefficient for the interaction term indicates whether the risk of household food hardship differed for income-eligible households that did and did not participate in SNAP prior to implementation of emergency allotments. Results suggested that the parallel trends assumption was met, conditional on potential confounders (RR for interaction term=0.97, 95% CI 0.92, 1.02 overall; RR for interaction term=1.02, 95% CI 0.95, 1.09 for households with Black children; RR for interaction term=0.99, 95% CI 0.94, 1.04 for households with Hispanic children; RR for interaction term=1.00, 95% CI 0.97, 1.03 for households with White children).

© 2024 Austin AE et al. *JAMA Network Open.*

eTable 2. Prevalence of Food Hardship in the Past 12 Months Among Households That Did and Did Not Participate in the Supplemental Nutrition Assistance Program (SNAP) and Had Incomes ≤130% of the Federal Poverty Level by Year (N= N=44,753)

	Households participating in the Supplemental Nutrition Assistance Program (SNAP) (N=20,474)		Households not participating in the Supplemental Nutrition Assistance Program (SNAP) (N=24,279)	
	**N**	**% (95% CI)**	**N**	**% (95% CI)**
2016	2,133	62.9 (59.7, 66.1)	1,639	44.3 (40.9, 47.8)
2017	1,019	58.9 (54.1, 63.6)	770	37.8 (33.3, 42.4)
2018	1,438	57.9 (53.9, 62)	1,197	40.7 (36.9, 44.4)
2019	1,313	56.5 (52.3, 60.8)	1,074	46.3 (42.3, 50.3)
2020	1,866	54.7 (51.2, 58.3)	1,439	40.7 (37.1, 44.3)
2021	2,065	48.2 (45, 51.4)	1,605	38.9 (35.6, 42.2)
2022	2,362	58.0 (55.1, 60.8)	2,088	47.5 (44.6, 50.4)

© 2024 Austin AE et al. *JAMA Network Open.*

eTable 3. Sensitivity Analyses for Change in Risk of Food Hardship From Before (2016-2019) to During (2020-2022) Implementation of Emergency Allotments in the Supplemental Nutrition Assistance Program (SNAP) for Income-Eligible Households With Children Participating in SNAP Compared to Income-Eligible Households With Children Not Participating in SNAP, 2016-2022 National Survey of Children's Health

	Adjusted[a] difference-in-difference estimator RR for interaction term (95% CI)
**Food hardship among households with incomes ≤130% FPL, excluding states that ended emergency allotments in 2021 or 2022**	
Households participating in SNAP	0.89 (0.78, 0.98)
Households not participating in SNAP	1.00
**Food hardship among households with incomes ≤130% FPL, excluding 2020**	
Households participating in SNAP	0.88 (0.80, 0.96)
Households not participating in SNAP	1.00
**Food hardship among households with incomes ≤130% FPL, excluding 2019**	
Households participating in SNAP	0.86 (0.79, 0.94)
Households not participating in SNAP	1.00
**Food hardship among households with incomes ≤130% FPL, adjusting for state adoption of broad-based categorical eligibility (BBCE) policies**	
Households participating in SNAP	0.90 (0.83, 0.97)
Households not participating in SNAP	1.00
**Food hardship among households with incomes ≤200% FPL**	
Households participating in SNAP	0.92 (0.86, 0.98)
Households not participating in SNAP	1.00
**Food insufficiency among households with incomes ≤130% FPL**	
Households participating in SNAP	0.88 (0.68, 1.14)
Households not participating in SNAP	1.00

[a]Adjusted for household receipt of cash assistance from a government program in the past 12 months, employment status of adults in the household, household income relative to the federal poverty level, child race and ethnicity, and state minimum wage, refundable Earned Income Tax Credit rate, maximum Temporary Assistance for Needy Families benefit for a family of 3, and Medicaid expansion

© 2024 Austin AE et al. *JAMA Network Open.*

eTable 4. Household Income Relative to the Federal Poverty Level by Child Race and Ethnicity, 2016-2022 National Survey of Children's Health

	Household income 0-65% of the federal poverty level (N=20,652)		Household income 66-130% of the federal poverty level (N=26,137)	
	N	% (95% CI)	N	% (95% CI)
**Child race**				
Black	4,046	28.0 (26.7, 29.2)	17,795	19.3 (18.3, 20.4)
White	12,619	52.3 (50.8, 53.8)	3,314	60.7 (59.4, 62.0)
All other races	3,987	19.8 (18.5, 21.1)	5,028	20.0 (18.9, 21.1)
**Child ethnicity**				
Hispanic	4,855	38.5 (36.9, 40.0)	5,770	37.3 (35.9, 38.7)
Non-Hispanic	15,797	61.6 (60.0, 63.1)	20,367	62.7 (61.3, 64.1)

© 2024 Austin AE et al. *JAMA Network Open.*
