# Supplementary material for: Emergency Allotments in SNAP and Food Hardship Among Households With Children
Source: JAMA Netw Open. 2024 Aug 16;7(8):e2428680. doi: 10.1001/jamanetworkopen.2024.28680 (PMC11329880; doi:10.1001/jamanetworkopen.2024.28680)
Supplement: Supplement 2. — Data Sharing Statement [file jamanetwopen-e2428680-s002.pdf]

## Data Sharing Statement

### Data

**Data available:** No

### Additional Information

**Explanation for why data not available:** National Survey of Children's Health data are freely available via request (https://www.childhealthdata.org/help/dataset).
